# Heteroleptic Pt(II)-dithiolene-based Colorimetric Chemosensors: Selectivity Control for Hg(II) Ion Sensing

**DOI:** 10.3390/ma13061385

**Published:** 2020-03-19

**Authors:** Hyokyung Jeon, Hwahui Ryu, Inho Nam, Dong-Youn Noh

**Affiliations:** 1 Department of Chemistry, Seoul Women’s University, Seoul 01797, Korea; hyokyung3@kbsi.re.kr (H.J.); torlxhrl369@naver.com (H.R.); 2 School of Chemical Engineering and Materials Science, Institute of Energy Converting Soft Materials, Chung-Ang University, Seoul 06974, Korea

**Keywords:** Hg^2+^ ion detection, colorimetric cation chemosensors, functional end-group manipulation, high selectivity, (diphosphine)Pt(dmit)

## Abstract

Hg^2+^ ions can accumulate in the natural environment and in organisms, where they cause damage to the central nervous system. Therefore, the detection of Hg^2+^ ions is essential for monitoring environmental contamination and human health. Herein, we demonstrate a simple method for tuning chemosensor signal ratios that significantly increased chemosensor selectivity for Hg^2+^ detection. Selectivity tuning was accomplished for chemosensors of the type (diphosphine)Pt(dmit), bearing the two different terminal groups 1,2-bis(diphenylphosphino)ethane (dppe) and 1,2-bis[bis(pentafluorophenyl)phosphino]ethane) (dfppe) due to the modulation of specific intermolecular interactions between the dmit ligand and Hg^2+^ ion. The structure exhibited a large pseudo-Stokes shift, which was advantageous for the internal reference signal and for eliminating potential artifacts. Straightforward chain-end manipulation enabled the tuning of chemosensor properties without additional chemical alterations. Based on these findings, we propose a new platform for improving the selectivity and sensitivity of colorimetric cation sensors. The results of this study will facilitate the designing of organic materials whose certain properties can be enhanced through precise control of the materials’ chemical hybridization by simple functional end-group manipulation.

## 1. Introduction

Ionic Hg (Hg^2+^) is well known for its high toxicity, and can be found in water, soil, and food [1]. The ions accumulate in the organism and interact with thiol groups of proteins, causing serious damage to the central nervous system and posing a significant threat to human health and the natural environment. Therefore, the detection of Hg^2+^ ions is a fundamental requirement for monitoring the environment and human health [2]. Various sensor platforms for Hg^2+^ detection have been developed to date, based on inorganic substances, oligonucleotides, liposomes, proteins, polymers, DNA glymes, small fluorescent organic molecules, and other compounds [2,3,4,5,6,7,8,9]. In supramolecular chemistry, the fluorescent chemosensor is one of the most significant designs because of the possibility of straightforward manipulation of photophysical processes that enables sensitive and selective signaling of targeted ion components [10]. Previously, we have reported a series of studies regarding (diphosphine)Pt(dmit) complexes (dmit: 1,3-dithiole-2-thione-4,5-dithiolate) as cation chemosensors that can be converted to operate via a ratiometric internal charge transfer, especially for Hg^2+^ detection [11,12,13]. These complexes created a large pseudo-Stokes shift between the analyte-free and analyte-bound emissive states. It rendered them wavelength-ratiometric, which means that potential artifacts were eliminated based on the internal referencing of the signal. Selectivity can be achieved only when the emission peaks for the free and bound-chemosensor shows clearly distinct intensities. However, the ratio of intensities is related to an inherent characteristic of a particular analyte and chemosensor interaction; hence, a systematic methodology for the control of signal ratio has rarely been practiced hitherto [10].

Herein, we demonstrate a simple method for chemosensor signal ratio tuning that significantly increased the chemosensor selectivity for Hg^2+^ detection. In order to achieve and explore signal ratio tuning of emission intensities, we set out to synthesize (dxpe)Pt(dmit) complexes with diphosphine-groups where dxpe = dppe (1,2-bis(diphenylphosphino)ethane) and dfppe (1,2-bis[bis(pentafluorophenyl)phosphino]ethane), while all other chemical functionalities remained constant. Both materials contained π-electrons in the >C=S double bond and a lone pair of electrons on the S atom; only the diphosphine end-groups were altered to obtain distinct electronegativities. A direct comparison of these two samples was made to elucidate the relationship between embedded terminal groups and the tunability of the sensing probe of the colorimetric sensors, based primarily on their electronic affinities and interfacial interactions with analyte cations. To the best of our knowledge, this is the first experimental report demonstrating the successful tuning of chemosensor properties by chain-end manipulation, without requiring further chemical alterations. We, therefore, propose a new platform for the improvement of selectivity of colorimetric cation sensors. This systematic investigation of end-functionalized chemicals serves to broaden the applicability and understanding of polymer science and provides new insights into advanced sensor technologies.

## 2. Materials and Methods

### 2.1. Synthesis

*Synthesis of (dfppe)PtCl*_2_. A dichloromethane (DCM) solution (30 mL) of (COD)PtCl_2_ (1.0 mmol, 374 mg) was added to an acetone solution (80 mL) of dfppe (1.0 mmol, 758 mg) and stirred overnight at room temperature. Following solvent removal, the solid was dissolved in methanol, filtered, and dried in air to yield 96.5% (988 mg) of the final product. Fourier Transform Infrared Spectroscopy (FT-IR) (KBr, cm^−1^) 2958, 2905 (C–H str.), 1646, 1524, 1482 (Ar C=C), 1100 (P–Ph), 982 (Ar C–F str.), and 538 (Ar ring oop def).

*Synthesis of (dfppe)Pt(dmit)*. A DCM solution (30 mL) of (dfppe)PtCl_2_ (0.5 mmol, 512 mg) and an acetone solution (30 mL) of *n*-Bu_2_Sn(dmit) (0.5 mmol, 214 mg) were mixed and stirred overnight at room temperature under an Ar atmosphere [14,15]. Evaporation of the solvent to 5 mL, followed by the addition of DCM, precipitated the red solid under vacuum. Following filtration and washing with DCM, the product was recrystallized from DCM/MeOH by slow diffusion, yielding 65.0% (374 mg) of a red product. Mp. > 278 °C (decomp.). EA cal. (obs.) for C_29_H_4_F_20_P_2_PtS_5_: C 30.30 (30.37); H 0.35 (0.39); S 13.95 (14.64). FT-IR (KBr, cm^−1^) 1644, 1522, 1478 (Ar C=C), 1101 (P–Ph), 1054 (C=S), 980 (Ar C–F str.), and 536 (Ar ring o.o.p. def).

*Synthesis of (dppe)Pt(dmit)*. The (dppe)Pt(dmit) complex was prepared using (dppe)PtCl_2_ and Na_2_(dmit) according to a previously reported procedure, and its spectroscopic data were in good agreement with the reported values [13].

### 2.2. General Characterization

Elemental analysis was performed using a FLASH2000 (Thermo Fisher Scienctific, Waltham, MA, USA) and PerkinElmer 2400 Series II installed at the National Center for Inter-University Research Facilities (NCIRF) at Seoul National University, Seoul, Korea. The infrared spectra were recorded using KBr pellets on a Perkin Elmer Spectrum 100 in the range of 400 ~ 4000 cm^−1^. UV-Vis absorption spectra were obtained on an S-3100 Photodiode Array spectrophotometer (SCINCO CO., Ltd., Seoul, Korea), using an H_2_O/CH_3_CN mixed solution (1:1 v/v %).

### 2.3. Electrochemical Analysis

Cyclic voltammetry measurements were conducted at room temperature with a computer-controlled potentiostat (CHI 620A Electrochemical Analyzer, CHI Instrument Inc., Bee Cave, TX, USA), using a standard three-electrode cell system with a Pt-button working electrode, a Pt wire as a counter electrode, and saturated Ag/AgCl as a reference electrode. Cyclic voltammograms were obtained in a DCM solution with 1.0 mM sample and 0.1 M n-Bu_4_N·ClO_4_ supporting electrolyte at a scan rate of 100 mV s^−1^. Ferrocene was used as a calibrant after each set of measurements, and all potentials reported were quoted with reference to the Fc/Fc^+^ redox couple (*E*_1/2_ = 0.532 V).

### 2.4. X-ray Diffraction (XRD) for Molecular Structure Analysis

Crystal structure analysis was performed by single-crystal diffraction methods at the Korea Basic Science Institute (KBSI, Western Seoul Center, Seoul, Korea). A single needle-like crystal (0.23 × 0.16 × 0.08 mm^3^) of (dfppe)Pt(dmit) was taken up with paratone oil and mounted on a Bruker SMART CCD diffractometer equipped with a graphite-monochromated Mo Kα (*λ* = 0.71073 Å) radiation source and a nitrogen cold stream (200(2) K). Data collection and integration was performed on a SMART (Bruker, Madison, WI, USA, 2000) and SAINT-Plus (Bruker, 2001) [16]. Absorption correction was performed by a multiscan method implemented in SADABS [17]. The structure was solved by direct methods and refined by full-matrix least-squares on *F*^2^ using SHELXTL [18]. All the nonhydrogen atoms were refined anisotropically, and hydrogen atoms were added to their geometrically ideal positions.

### 2.5. Density Functional Theory Calculations

**Figure 1:**
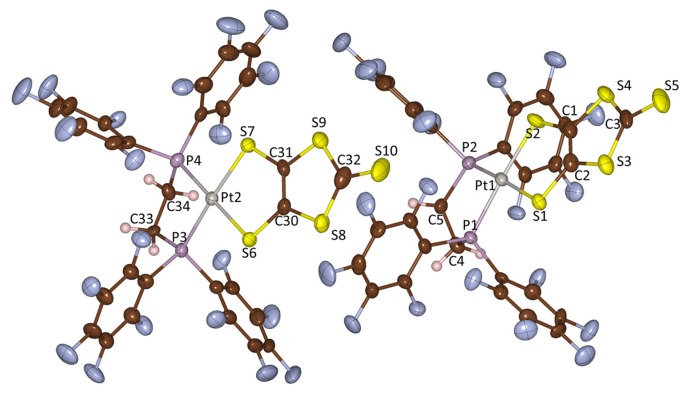
Molecular structure of (dfppe)Pt(dmit) with the selected numbering scheme. Bond lengths (Å) and angles (°): Pt1-P1 2.2473(18), Pt1-P2 2.251(2), Pt1-S1 2.3023(19), Pt1-S2 2.3094(19), Pt2-P3 2.2554(19), Pt2-P4 2.2549(19), Pt2-S6 2.3107(19), Pt2-S7 2.3094(18), C1-C2 1.347(11), C3-S5 1.650(8), C30-C31 1.349(11), C32-S10 1.648(8), P1-Pt1-P2 86.59(7), P2-Pt1-S2 90.05(7), P1-Pt1-S2 175.51(7), P2-Pt1-S1 172.59(7), P1-Pt1-S1 90.38(7), S1-Pt1-S2 90.45(7), P3-Pt2-P4 86.48(7), P3-Pt2-S6 91.64(7), P4-Pt2-S6 175.93(7), P3-Pt2-S7 172.11(7), P4-Pt2-S7 91.51(7), and S6-Pt2-S7 89.88(7).

First-principles calculations were carried out on the basis of density functional theory (DFT) using a generalized gradient approximation (GGA) within the Perdew–Burke–Ernzerhof (PBE) functional [19,20]. We used the projector-augmented wave (PAW) method for describing ionic cores as implemented in the Vienna ab initio simulation package [21]. A plane-wave basis set with a kinetic-energy cut-off of 600 eV and a 4 × 4 × 4 Monkhorst–Pack k-point mesh was used. The electronic optimization steps were converged self-consistently over 10^−4^ eV per formula unit. The cation adsorbed on each (dxpe)Pt(dmit) in the most stable state was directly relaxed by energy optimization from any initial positions. The charge accumulations and charge reductions around the molecules are shown in Figure 1, as yellow and blue surfaces, respectively. In this study, the unit of charge densities was e *a*_0_^−3^, where e was the elementary charge and *a*_0_ was the Bohr radius.

## 3. Results and Discussion

The selective metal ion-sensing properties of (dxpe)Pt(dmit) complexes were demonstrated by unique color changes. To validate the selectivity of the chemosensors in practice, a series of metal cations were added to a solution of the chemosensor under identical conditions. The protocol was as follows: (1) the materials analyzed were fabricated with differing end-functional groups and (2) the color changes were observed with the naked eye and by UV-visible spectrometry.

### 3.1. Synthesis and XRD for Structural Analysis

**Scheme 1:**
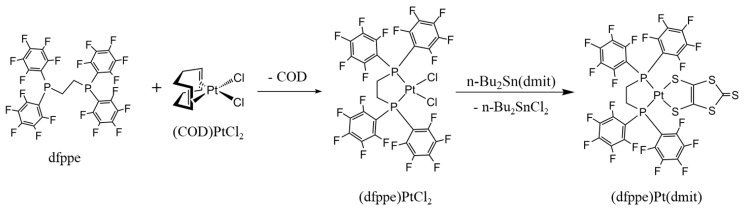
Synthesis of (dfppe)Pt(dmit).

The (dppe)Pt(dmit) complex was prepared from (dppe)PtCl_2_ and Na_2_(dmit) according to a previously reported procedure [12]. The synthesis of the (dfppe)Pt(dmit) complex, having an F-enriched end-functional group, involved (dfppe)PtCl_2_ and *n*-Bu_2_Sn(dmit), following an analogous salt elimination process (Scheme 1). Product identification was confirmed by spectroscopic analyses. The structure of the as-synthesized (dfppe)Pt(dmit) complex was additionally confirmed by X-ray diffraction for molecular structure analysis (Table 1 and Table 2, and Figure 1). The molecular structure of (dfppe)Pt(dmit) shown in Figure 1 is virtually isostructural with that of (dppe)Pt(dmit) [22], with the former bearing F, and the latter H atoms on the diphosphine chelate ligands. Their principal bond lengths and angles were likewise analogous, as compared in Table 1.

### 3.2. Electrochemical Analysis

**Figure 2:**
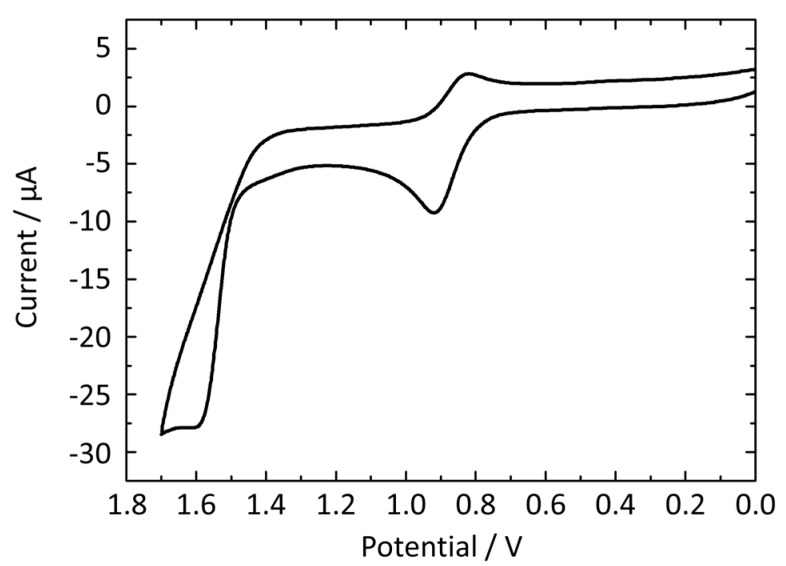
Cyclic voltammogram of (dfppe)Pt(dmit) measured in dichloromethane (DCM) (Fc/Fc^+^ = 0.532 V *vs* Ag/AgCl, 0.1 M *n*-Bu_4_N⋅ClO_4_, Pt-button electrode, Ag/AgCl, 100 mV/s).

The (dxpe)Pt(dmit) complexes (dxpe = dppe and dfppe) exhibited identical cyclic voltammograms with one reversible cycle at a lower potential region and one irreversible oxidation peak at a higher region. However, their redox potential values were not identical due to differences in the donor capability of the dithiolate ligand, which is affected by the diphosphine ligand. The cyclic voltammogram of (dfppe)Pt(dmit) exhibits a reversible cycle at $E_{1/2}^{1}$ = 0.871 V ($E_{pa}^{1}$ = 0.921 V and $E_{pc}^{1}$ = 0.820 V), and an irreversible anodic peak at $E_{pa}^{2}$ = 1.600 V (Figure 2). This half-wave potential can be attributed to the redox process of the Pt(dmit) moiety [23], which suggests that [(dfppe)Pt(dmit)]^+^, formed during the first redox process at $E_{1/2}^{1}$ = 0.871 V, is well stabilized by the Pt(dmit) moiety. This potential is higher than that of (dppe)Pt(dmit) ($E_{1/2}^{1}$ = 0.777 V), indicating that the F-ended functional groups of (dfppe)Pt(dmit) decreased the donor capability of the Pt(dmit) moiety compared to H-ended functional groups of (dppe)Pt(dmit). This variance is likely to endow distinct sensing properties to these chemosensors.

### 3.3. Colorimetric Sensing Properties

**Figure 3:**
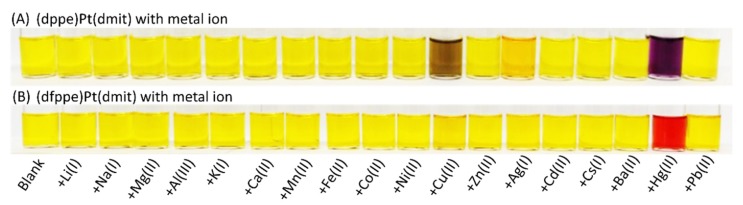
Color changes of H_2_O/CH_3_CN solution (0.2 mM, 1:1 v/v %) of (**A**) (dppe)Pt(dmit) and (**B**) (dfppe)Pt(dmit) upon the addition of one equivalent of metal ions.

To compare the selectivity of the chemosensors, 18 commercially available metal perchlorate compounds were considered (Figure 3). Both chemosensors exhibited the most efficient sensing capabilities for the Hg^2+^ ion, but their selectivity differed significantly based on the nature of the end-functional group. The color change of the chemosensor in H_2_O/CH_3_CN solution (0.2 mM, 1:1 v/v %) with various metal cations is clearly depicted in Figure 3. Both (dfppe)Pt(dmit) and (dppe)Pt(dmit) complexes in aqueous solution exhibited virtually no color change with hard acids by Pearson’s acid-base concept, such as Na^+^, K^+^, and Ca^2+^ [24]. These observations are understandable, as coordination between hard Lewis acids and thione moiety (>C=S) is unfavorable. The dmit ligand is an electron-rich moiety, containing π-electrons in the >C=S double bond and a lone pair of electrons on the S atom; therefore, it is categorized as a soft base. On the other hand, the binding of several cations, such as Cu^2+^ and Ag^+^, occurs with the (dppe)Pt(dmit) chemosensor, in which the cation receptor is an electron donor as well as part of the π-system of the chromophore. Cu^2+^ and Ag^+^ ions are soft acids and can thus chelate weakly with the thione moiety. Therefore, although the response was minimal compared to that observed for the Hg^2+^ cation, a color change with a blue shift was observed for Cu^2+^ and Ag^+^ in an aqueous solution of (dppe)Pt(dmit), even by naked eye, as shown in Figure 3A.

It is worth noting the selective sensing properties of the (dppe)Pt(dmit) complex toward Hg^2+^, Cu^2+^ and Ag^+^ cations among the 18 metal ions, in light of the standard reduction potentials (*E*°) of the metal ions. The three metal cations have positive *E*° values (*E*° = +0.854 V for Hg^2+^/Hg, +0.34 V for Cu^2+^/Cu, and +0.80 V for Ag^+^/Ag at 298 K), while the values of the other 15 metal cations are negative [25]. Therefore, during the sensing process, the three metal cations with positive reduction potentials would be more susceptible to reduction and would, consequently, withdraw electrons more readily from the dmit ligand, inducing the color change in the chemosensor.

Altering the diphosphine end-group of the chemosensor from dppe to dfppe dramatically influenced its selectivity for metal ions. Notably, alkali metals, alkaline earth metals, and transition metal ions did not induce any apparent color change, including Cu^2+^ and Ag^+^ cations. Of the 18 metal cations tested, only Hg^2+^ ions induced a color change with the (dfppe)Pt(dmit) chemosensor, from yellow to vivid red (Figure 3B). This suggests that the interaction between the >C=S moiety and metal ions was dependent on the properties of the end-functional group of the chemosensor. That is, the F-enriched sensor, which has the highest reduction potential among the 18 metal ions, exhibited excellent selectivity for Hg^2+^ detection.

### 3.4. Absorption Spectroscopy on Sensing of Metal Ions

**Figure 4:**
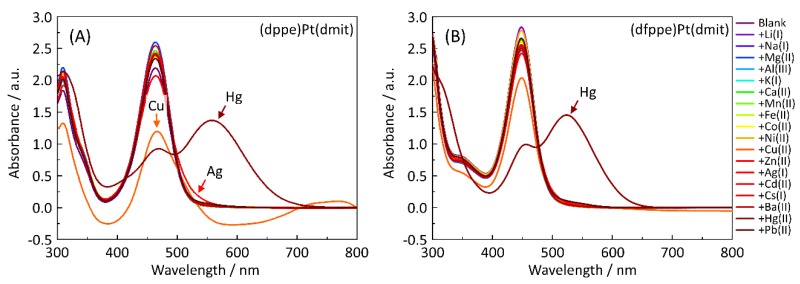
UV-vis spectra of (**A**) (dppe)Pt(dmit) and (**B**) (dfppe)Pt(dmit) complexes with or without metal ions (Li^+^, Na^+^, K^+^, Cs^+^, Mg^2+^, Ca^2+^, Ba^2+^, Mn^2+^, Fe^2+^, Co^2+^, Ni^2+^, Cu^2+^, Zn^2+^, Ag^+^, Cd^2+^, Hg^2+^, Al^3+^, and Pb^2+^) dissolved in H_2_O/CH_3_CN solution (1:1 v/v %). The absorption peaks due to the >C=S moiety observed at (**A**) 463 nm and (**B**) 448 nm show a noticeable bathochromic shift upon the addition of Hg^2+^ ions.

**Figure 5:**
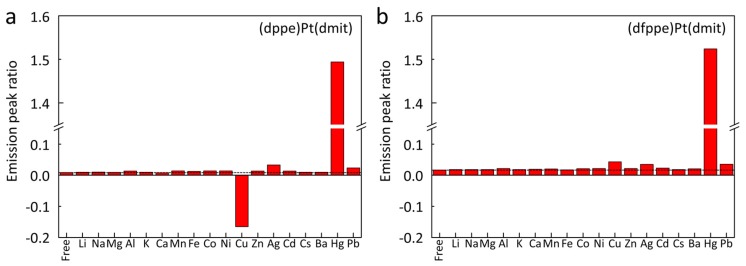
Absorbance ratios (A_558_/A_464_ and A_524_/A_448_) from the UV-Vis spectra of (**a**) (dppe)Pt(dmit) and (**b**) (dfppe)Pt(dmit), respectively, with or without metal ions dissolved in H_2_O/CH_3_CN solution (1:1 v/v %).

The Hg^2+^ sensing properties of the (dxpe)Pt(dmit) complexes were quantitatively investigated by monitoring the shift in their absorption peak by UV-visible spectrometry, which corresponds to the shift of the π→π* transition of the >C=S moiety in the dmit ligand (Figure 4 and Figure 5). In the absence of cations, the absorption spectra of (dppe)Pt(dmit) and (dfppe)Pt(dmit) contained one peak of medium intensity at λ_1_ = 463 nm and λ_2_ = 448 nm, respectively. The peaks in the spectra resemble those of complexes containing the Pt(dmit) moiety, such as (Ph_3_P)_2_Pt(dmit) and (P2)Pt(dmit) (P2 = 3,4-dimethyl-3’,4’-bis(diphenylphosphino)tetrathiafulvalene), indicating that they originated from the absorption of the >C=S moiety [23]. As various metal cations were added to the yellow solution of (dppe)Pt(dmit), the absorption intensity at λ_1_ decreased, with concurrent evolution of new peaks for the three metal ions (Hg^2+^, Cu^2+^, and Ag^+^) in the higher wavelength region: the characteristic bathochromic shift to 558 nm for Hg^2+^, a shift to 700–800 nm for Cu^2+^, and a slight increase (and consequently, a weak color change in Figure 3A) at approximately 530 nm for Ag^+^. In the case of the (dfppe)Pt(dmit) chemosensor, however, only the Hg^2+^ ion led to the evolution of a new absorption peak at 523 nm with a decrease in intensity at λ_2_. This bathochromic shift was accompanied by a color change of the solution to vivid red, as seen in Figure 3.

### 3.5. DFT Calculation

**Figure 6:**
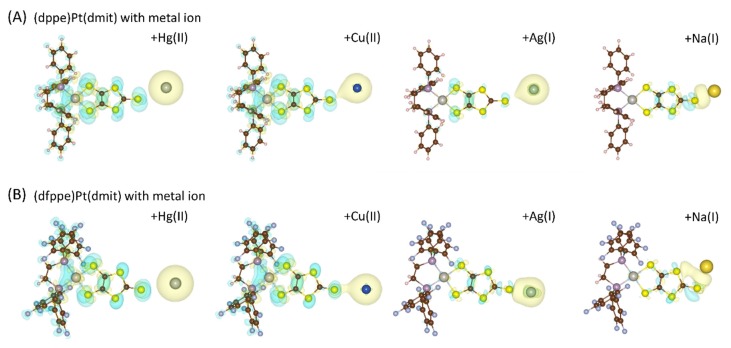
Charge analyses of (**A**) (dppe)Pt(dmit) and (**B**) (dfppe)Pt(dmit) complexes with representative metal ions. The charge accumulations and charge reductions around the molecules are shown as yellow and blue surfaces, respectively. The isosurface is 0.0015e *a*_0_^−3^, where *a*_0_ is the Bohr radius.

To rationalize the differential selectivity between (dppe)Pt(dmit) and (dfppe)Pt(dmit) for Hg^2+^ chemosensing, we investigated the mechanism involved in the interaction between the reactants at the atomic level using DFT calculations. The exposed S with a lone pair of electrons on the >C=S moiety had effective charges of 5.11e and 4.87e for (dppe)Pt(dmit) and (dfppe)Pt(dmit), respectively. The difference in these values was caused by the differing electronegativities of H(2.1) and F(4.0) protruding from the diphosphine ligands, which have an average effective charge of 0.95e and 7.81e, respectively. In the plots of differential charge densities shown in Figure 6, both cases exhibit a hybridized region between the chemosensor and the metal ion via intermolecular >C=S···***M*** (***M***: metal ion) interactions in the solvent, indicating the occurrence of chemical adhesion. The differential charge density, *ρ*_diff_, of >C=S···***M*** adhesion is defined by

*ρ*_diff_ = *ρ*_total_ − (*ρ*_(dxpe)Pt(dmit)_ + *ρ_M_*)

where *ρ*_total_ is the charge density of S···***M*** adhesion, *ρ*_(dxpe)Pt(dmit)_ is the charge density for a single (dxpe)Pt(dmit) unit, and *ρ_M_* is the charge density of the metal ions [26]. Charge accumulation (yellow) and charge reduction (blue) around (dxpe)Pt(dmit) and the metal ions are explicitly shown in Figure 6.

The two distinct charge densities of the (dxpe)Pt(dmit) chemosensors share a common feature: charge accumulation and reduction were horizontally superposed between the metal ions(***M***) and the S of the dmit moiety, which suggests an electrostatic interaction at the >C=S···***M*** site. In this molecule, the interaction energy was dependent on the nature of the metal ion. In the case of Hg^2+^ ions, electronic hybridization significantly increased. On the other hand, hybridization decreased in the order of Ag^+^, Cu^2+^, and Na^+^, which is in good agreement with the results of the colorimetric sensing property analysis of (dxpe)Pt(dmit). The interaction characteristics between (dppe)Pt(dmit) and (dfppe)Pt(dmit) were analogous; however, their absolute values varied significantly. The variance in the effective charge on the S atom of the dmit moiety, with and without various metal ions, is shown in Table 3. The (dfppe)Pt(dmit) complex exhibited a notably smaller variance compared to (dppe)Pt(dmit), based on the all-metal ions. It should be noted that the selectivity of the chemosensor for other cations can be controlled by the hybridization shielding effect of F-ended functional groups.

## 4. Conclusions

We presented a novel methodology for the enhancement of Hg^2+^ detection by the introduction of differing functional terminal groups on (diphosphine)Pt(dmit) chemosensors. The use of distinct terminal groups enabled selectivity tuning, which is attributed to the modulation of specific intermolecular interactions. Organization of the end groups on the ligand moiety proved beneficial for altering whole-material properties. The results hold implications for the design of organic materials, wherein crucial properties can be enhanced through precise control of their chemical hybridization by functional end-group manipulation.

## Figures and Tables

**Table 1:** Crystal data and structure refinement parameters for (dfppe)Pt(dmit).

Empirical Formula	C_29_ H_4_ F_20_ P_2_ Pt S_5_
Formula weight	1149.65
Crystal system	Monoclinic
Space group	P2(1)/n
*a* (Å)	12.2555(10)
*b* (Å)	13.7690(11)
*c* (Å)	40.429(3)
*β* (°)	90.906(2)
Volume (Å^3^)	6821.4(9)
Z	8
Density Cal. (Mg/m^3^)	2.239
Absorption coefficient (mm^−1^)	4.650
*F*(000)	4368
Crystal size (mm^3^)	0.23 × 0.16 × 0.08
Theta range for data collection (°)	1.007 to 26.017
Index ranges	−15 ≤ *h* ≤ 8, −16 ≤ *k* ≤ 16, −49 ≤ *l* ≤ 46
Reflections collected	41778
Independent reflections	13361 [R(int) = 0.0618]
Completeness to theta = 25.242° (%)	99.8
Absorption correction	Semiempirical from equivalents
Refinement method	Full-matrix least-squares on *F*^2^
Data/restraints/parameters	13361/0/1027
Goodness-of-fit on *F*^2^	1.037
Final R indices [*I* > 2σ(*I*)]	*R*1 = 0.0442, w*R*2 = 0.0938
R indices (all data)	*R*1 = 0.0757, w*R*2 = 0.1147
Largest diff. peak and hole (e.Å^−3^)	2.062 and −1.187

**Table 2:** Comparison of selected bond lengths (Å) and angles (°) between (dppe)Pt(dmit) and (dfppe)Pt(dmit).

Complexes	Bond Lengths (Å)				Bond Angles (°)	
	Pt1-P1	Pt1-P2	Pt1-S1	Pt1-S2	P1-Pt-P2	S1-Pt-S2
(dppe)Pt(dmit) ^1)^	2.251(3)	2.258(3)	2.315(3)	2.308(3)	85.2(1)	90.0(1)
(dfppe)Pt(dmit)	2.2473(18)	2.251(2)	2.3023(19)	2.3094(19)	86.59(7)	90.45(7)
	2.2554(19)	2.2549(19)	2.3107(19)	2.3094(19)	86.48(7)	89.88(7)

^1)^ Ref. [22].

**Table 3:** Bader atomic charge analyses for the effective charges of (dppe)Pt(dmit) and (dfppe)Pt(dmit) complexes with and without representative metal ions.

Complexes	−e ^1)^	Δ(−e) ^2)^			
		Hg^2+^	Cu^2+^	Ag^+^	Na^+^
(dppe)Pt(dmit)	5.11	−1.76	−1.42	−0.66	−0.08
(dfppe)Pt(dmit)	4.87	−1.73	−1.18	−0.35	−0.08

^1)^ Effective charge of S on >C=S moiety in (dxpe)Pt(dmit) without metal ions; ^2)^ Differential effective charge of S on >C=S moiety in (dxpe)Pt(dmit) with metal ions.

## References

[1] Valko M., Morris H., Cronin M.T.D. (2005). Metals, toxicity and oxidative stress. Curr. Med. Chem..

[2] Guo C., Irudayaraj J. (2011). Fluorescent ag clusters via a protein-directed approach as a Hg(II) ion sensor. Anal. Chem..

[3] Chen P., He C.A. (2004). A General strategy to convert the merr family proteins into highly sensitive and selective fluorescent biosensors for metal ions. J. Am. Chem. Soc..

[4] Chen Y.Q., Bai H., Hong W.J., Shi G.Q. (2009). Fluorescence detection of mercury ions in aqueous media with the complex of a cationic oligopyrene derivative and oligothymine. Analyst.

[5] Huang C.-C., Yang Z., Lee K.-H., Chang H.-T. (2007). Synthesis of highly fluorescent gold nanoparticles for sensing mercury(II). Angew. Chem. Int. Ed..

[6] Li D., Wieckowska A., Willner I. (2008). Optical analysis of Hg^2+^ ions by oligonucleotide-gold-nanoparticle hybrids and DNA-based machines. Angew. Chem. Int. Ed..

[7] Wang Z.D., Lee J.H., Lu Y. (2008). Highly sensitive “turn-on” fluorescent sensor for Hg^2+^ in aqueous solution based on structure-switching DNA. Chem. Commun..

[8] Yigit M.V., Mishra A., Tong R., Cheng J.J., Wong G.C.L., Lu Y. (2009). Inorganic mercury detection and controlled release of chelating agents from ion-responsive liposomes. Chem. Biol..

[9] Yoon S., Miller E.W., He Q., Do P.H., Chang C.J. (2007). A bright and specific fluorescent sensor for mercury in water, cells, and tissue. Angew. Chem. Int. Ed..

[10] Coskun A., Akkaya E.U. (2006). Signal ratio amplification via modulation of resonance energy transfer: Proof of principle in an emission ratiometric Hg(II) sensor. J. Am. Chem. Soc..

[11] Nam H.J., Lee H.-J., Noh D.-Y. (2004). Novel mercury(II) complexes of 1,3-dithiole-2-thiones containing the 2-pyridyl moiety: Syntheses, X-ray crystal structures and solution behavior. Polyhedron.

[12] Jeon S., Suh W., Noh D.-Y. (2017). Anion-dependent Hg^2+^-sensing of colorimetric (dppe)Pt(dmit) chemosensor (dppe: 1,2-bis(diphenylphosphino)ethane; dmit: 1,3-dithiole-2-thione-4,5-dithiolate). Inorg. Chem. Commun..

[13] Jeon H., Suh W., Noh D.-Y. (2012). Hg(II) sensing properties of (diphosphine)Pt(dmit) complexes (dmit: C_3_S_5_^2−^: 1,3-dithiole-2-thione-4,5-dithiolate). Inorg. Chem. Commun..

[14] Nomura M., Fourmigué M. (2008). Dinuclear Cp* Cobalt Complexes of the 1,2,4,5-Benzenetetrathiolate Bischelating Ligand. Inorg. Chem..

[15] Doidge-Harrison S.M., Irvine J.T., Khan A., Spencer G.M., Wardell J.L., Aupers J.H. (1996). Diorganotin 1,3-dithiole-2-thione-4,5-dithiolate compounds, R2Sn(dmit): The crystal structure of MePhSn(dmit). J. Organomet. Chem..

[16] Bruker AXS Inc. (2000). SMART, SAINT-Plus v 6.22 and XPREP.

[17] Sheldrick G.M. (2002). SADABS v 2.03.

[18] Bruker AXS Inc. (2000). SHELXTL v 6.10.

[19] Perdew J.P., Burke K., Ernzerhof M. (1996). Generalized gradient approximation made simple. Phys. Rev. Lett..

[20] Kresse G., Furthmüller J. (1996). Efficient iterative schemes for ab initio total-energy calculations using a plane-wave basis set. Phys. Rev. B Condens. Matter Mater. Phys..

[21] Grimme S. (2006). Semiempirical GGA-Type density functional constructed with a long-range dispersion correction. J. Comput. Chem..

[22] Vicente R., Ribas J., Solans X., Font-Altaba M., Mari A., Loth P., Cassoux P. (1987). Electrochemical, EPR, and crystal structure studies on mixed-ligand 4,5-dimercapto-l,3-dithia-2-thione phosphine complexes of nickel, palladium and platinum, M(dmit)(dppe) and Pt(dmit)(PPh_3_)_2_. Inorg. Chim. Acta.

[23] Shin K.S., Jung Y., Lee S.K., Fourmigué M., Barrière F., Bergamini J.F., Noh D.Y. (2008). Redox bifunctionality in a Pt(II) dithiolene complex of a tetrathiafulvalene diphosphine ligand. Dalton Trans..

[24] Pearson R.G. (1963). Hard and soft acids and bases. J. Am. Chem. Soc..

[25] Rumble J. (2019). CRC Handbook of Chemistry and Physics.

[26] Bae S., Nam I., Park S., Yoo Y.G., Yu S., Lee J.M., Han J.W., Yi J. (2015). Interfacial adsorption and redox coupling of Li_4_Ti_5_O_12_ with nanographene for high-rate lithium storage. ACS Appl. Mater. Interfaces.

